# Implication of NPM1 phosphorylation and preclinical evaluation of the nucleoprotein antagonist N6L in prostate cancer

**DOI:** 10.18632/oncotarget.8043

**Published:** 2016-03-14

**Authors:** Damien Destouches, Maha Sader, Stéphane Terry, Charles Marchand, Pascale Maillé, Pascale Soyeux, Gilles Carpentier, Fannie Semprez, Jocelyn Céraline, Yves Allory, José Courty, Alexandre De La Taille, Francis Vacherot

**Affiliations:** ^1^ Université Paris-Est, UPEC, Créteil, F-94000, France; ^2^ INSERM, U955, Equipe 7, Créteil, F-94000, France; ^3^ CNRS, ERL 9215, Laboratoire de Recherche sur la Croissance Cellulaire, la Réparation et la Régénération Tissulaires (CRRET), Créteil, F-94000, France; ^4^ INSERM, U1186, Gustave Roussy Cancer Campus, Villejuif, F-94805, France; ^5^ AP-HP, Hôpital H. Mondor – A. Chenevier, Département de Pathologie, Créteil, F-94000, France; ^6^ AP-HP, Hôpital H. Mondor – A. Chenevier, Département d'Urologie, Créteil, F-94000, France; ^7^ INSERM, U1113, Fédération de Médecine Translationnelle de Strasbourg (FMTS), Université de Strasbourg, Strasbourg, F-67000, France

**Keywords:** prostate cancer, NPM1, phosphorylated NPM1, N6L, androgen receptor

## Abstract

Despite the advent of several new treatment options over the past years, advanced/metastatic prostate carcinoma (PCa) still remains incurable, which justifies the search for novel targets and therapeutic molecules. Nucleophosmin (NPM1) is a shuttling nucleoprotein involved in tumor growth and its targeting could be a potential approach for cancer therapy. We previously demonstrated that the multivalent pseudopeptide N6L binds to NPM1 potently affecting *in vitro* and *in vivo* tumor cell growth of various tumor types as well as angiogenesis. Furthermore, NPM1 binds to androgen receptor (AR) and modulate its activity. In this study, we first investigated the implication of the NPM1 and its Thr199 and Thr234/237 phosphorylated forms in PCa. We showed that phosphorylated forms of NPM1 interact with androgen receptor (AR) in nucleoplasm. N6L treatment of prostate tumor cells led to inhibition of NPM1 phosphorylation in conjunction with inhibition of AR activity. We also found that total and phosphorylated NPM1 were overexpressed in castration-resistant PCa. Assessment of the potential therapeutic role of N6L in PCa indicated that N6L inhibited tumor growth both *in vitro* and *in vivo* when used either alone or in combination with the standard-of-care first- (hormonotherapy) and second-line (docetaxel) treatments for advanced PCa. Our findings reveal the role of Thr199 and Thr234/237 phosphorylated NPM1 in PCa progression and define N6L as a new drug candidate for PCa therapy.

## INTRODUCTION

Prostate carcinoma (PCa) is currently the second most common malignancy and the sixth leading cause of cancer death among men worldwide [1]. Since work in the early 1940's by Charles Huggins identifying androgens as the source of PCa growth, the main treatment for metastatic PCa patients remains androgen withdrawal. Unfortunately, after an initial high clinical rate, tumors recur leading to a castration-resistant prostate cancer (CRPC) state. First line treatment is either a docetaxel-based chemotherapy that confers a modest survival improvement with less than six months' duration [2–4] or second line hormonal therapy with abiraterone acetate or enzalutamide due to persistent tissue levels of androgens and recurrent activity of androgen receptor (AR) [5–9]. Over the past years, several other new therapeutic agents including cabazitaxel, Sipuleucel-T and radium-223 have been also shown to confer a survival benefit to CRPC and chemoresistant patients [10] [11] [12]. Nevertheless, all these treatments remain palliative and research efforts are still needed to provide new therapeutic solutions.

Nucleophosmin (NPM1/B23/NO38/numatrin) is a multifunctional protein involved in several cellular processes including ribosome biogenesis [13], centrosome duplication [14], molecular chaperone activities [15] and nucleocytoplasmic protein trafficking [16]. NPM1 also plays important roles in cancer since it is over-expressed in proliferating cells compared to normal cells [17, 18] and has been proposed to be a marker of different solid tumors such as prostate [19], colon [20], ovarian [21], gastric [22], bladder [23], hepatomic [24], oral [25] and thyroid [26, 27] carcinomas. NPM1 can be phosphorylated by different kinases modifying its localization and functions (reviewed in [28]). Indeed, phosphorylation by CDK2/cyclin E on Thr199 residue during G1 phase is critical for the NPM1 dissociation from the centrosome to initiate its duplication and the pre-mRNA processing [29]. NPM1 is also phosphorylated on threonine residues (Thr199, Thr219 and Thr234/237) during mitosis allowing its dissociation from the nucleolus [30].

We previously demonstrated that the multivalent pseudopeptide N6L binds to two major nucleoproteins, nucleolin and NPM1, and inhibited tumor growth and associated angiogenesis [31]. Recently, it has been demonstrated that NPM1 could bind to AR thereby modulating its activity [32]. In the present study, we explored in which cell compartment the AR/NPM1 interaction could occur and we evaluated the effect of N6L on NPM1 phosphorylation and AR activity. We then studied the expression levels of total NPM1 and two of its phosphorylated forms, Thr199 and Thr234/237, in different stages of human PCa. Finally, we evaluated the effect of N6L on prostate tumor growth, *in vitro* and *in vivo*, alone or in combination with either hormonal therapies or docetaxel treatments.

## RESULTS

### Phosphorylated Thr199 and Thr234/237 forms of NPM1 and AR were both localized in sub-nuclear structures

**Figure 1:**
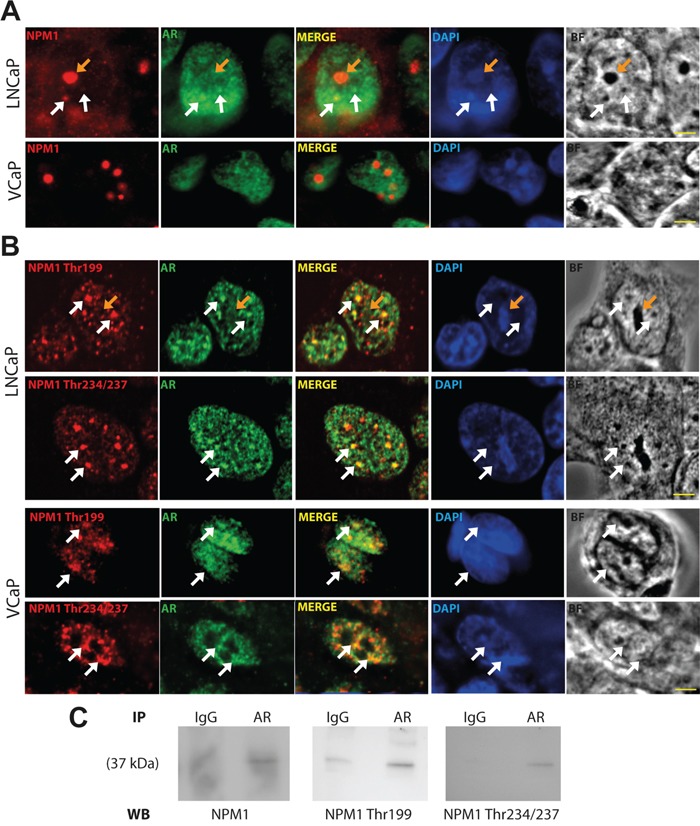
AR and Thr199 and Thr234/237 NPM1 localized in sub-nuclear structures **A** and **B.** LNCaP and VCaP cells were starved for 24 hours and then stimulated with 10 nmol/L DHT for 1 hour. Cells were fixed with methanol, immunostained (NPM1, AR and Thr199 and Thr234/237 NPM1) and analyzed by spinning disk fluorescence microscopy. Residual blurring was removed by spatial deconvolution. Nuclei were stained with DAPI. Scale bars, 2.5 μm. White arrows show the co-immunofluorescences and orange arrows show nucleoli. **C.** Nuclei of stimulated LNCaP were purified and AR was immuno-precipitated. The presence of NPM1 and its two phosphorylated forms were revealed by Western blotting.

It has been demonstrated that NPM1 interacts with AR and modulates its activity [32] but in which cellular compartment the AR/NPM1 interaction may occur has yet to be determined. To answer this question, we performed immunofluorescence analyses and examined the localization of these two proteins in two dihydrotestosterone (DHT) responsive cell lines, LNCaP and VCaP. NPM1 was mainly localized in nucleolus and AR was mainly localized in nucleoplasm of both cell lines showing no colocalization (Figure 1A). Nevertheless, some spots of coincidental staining were observed in the nucleoplasm of LNCaP cells, while expectedly, such spots were absent in VCaP cells as NPM1 is expressed at marginal level in the nucleoplasm. Nucleoplasmic NPM1 displays the particularity to be phosphorylated [33], we next asked if the two phosphorylated forms of NPM1, Thr199 and Thr234/237, could colocalize with AR. In interphasic LNCaP and VCaP, the two phosphorylated forms were absent of the nucleoli and localized in sub-nuclear structures, named nuclear speckles, that appeared dense in bright field pictures and were stained with the anti-sc-35 antibody (Figure 1B and Supplementary Figure 1C). Furthermore, as already described [34], high expression of Thr199 and Thr234/237 NPM1 was noted during mitosis in LNCaP and VCaP cells with an increase in expression until metaphase followed by a decrease from anaphase to interphase (Supplementary Figure 2). Interestingly, AR was also observed in these sub-nuclear structures in LNCaP and VCaP cells. The two-labeled spots from AR and phosphorylated NPM1 appeared juxtaposed and partially fused suggesting a probable contact between the two proteins in these nucleoplasm structures (Figure 1B). In addition, NPM1 and its two phosphorylated forms co-immunoprecipitate with AR arguing for the idea of an interaction between these two proteins (Figure 1C). These results suggest a role for the phosphorylated status of NPM1 in AR signaling and a potential role in PCa development.

### N6L treatment interferes with NPM1 phosphorylation and AR activity

**Figure 2:**
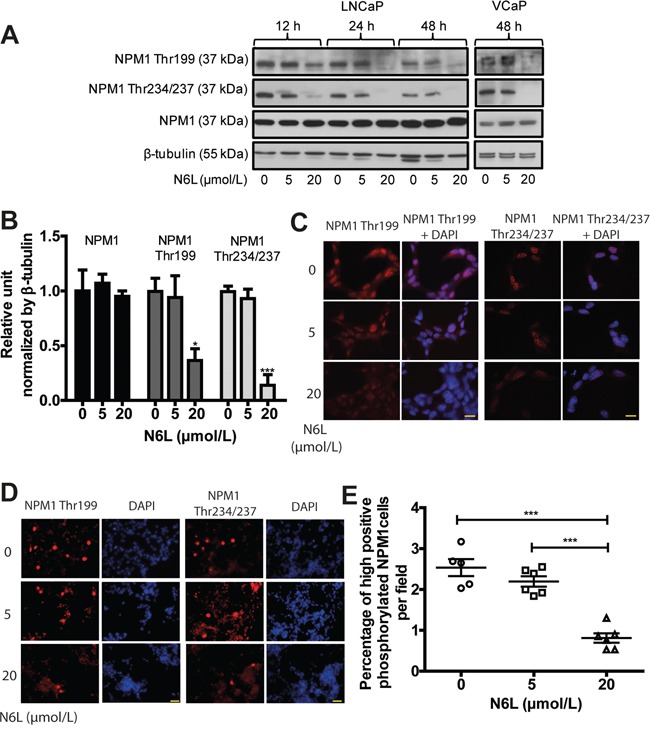
N6L reduced NPM1 phosphorylation on Thr199 and Thr234/237 LNCaP or VCaP cells were treated for 12, 24 or 48 hours with or not 5 or 20 μmol/L N6L. **A.** Protein expressions analyzed by Western blot. **B.** Densitometry quantification (ImageJ software) of protein expressions of LNCaP cells treated or not for 48 hours with N6L showed by protein/β-tubulin ratio density. Means ± sd (n = 3), * p < 0.05 and *** p < 0.001. **C** and **D.** LNCaP cells, treated or not with N6L for 48 hours, were fixed with methanol, immunostained (Thr199 and Thr234/237 NPM1) and analyzed by spinning disk fluorescence microscopy at high (C: scale bar, 5 μm) or low magnification (D: scale bar, 20 μm). Nuclei were stained with DAPI. **E.** Quantification of cells displaying strong staining of phosphorylated Thr199 or Thr234/237 NPM1. Data are expressed as the percentage of strong positive cells from 5 pictures per condition. Means ± sd (n = 5 for control and 6 for treated cells), *** p < 0,001.

N6L is an inhibitor of tumor growth and angiogenesis and one of its molecular targets is NPM1 [31]. As NPM1 phosphorylation seems to be involved in PCa development, we set out to investigate N6L effect on the expression of total and phosphorylated NPM1 using Western blotting experiments on LNCaP cells. Total NPM1 expression did not appear to change upon N6L treatment neither with time of treatment nor with the concentration used whereas a substantial decrease in NPM1 phosphorylation was observed with 20 μmol/L N6L (63% for Thr199 and 86% for Thr234/237 of reduction at 48 hours) (Figure 2A and 2B). Comparable results were observed in VCaP cells (Figure 2A). Immunofluorescent analysis showed that the reduction of NPM1 phosphorylation by N6L was due to a global decrease in phosphorylated NPM1 in interphasic cells associated with a decrease in the number of strongly stained mitotic cells as observed at a lower magnification (Figure 2C–2E). These results suggest that N6L effectively impedes NPM1 phosphorylation.

**Figure 3:**
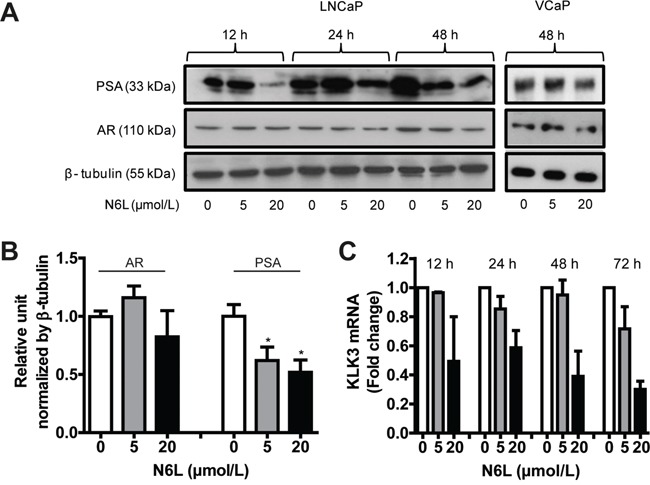
N6L inhibited PSA expression LNCaP or VCaP cells were treated or not for 12, 24 or 48 hours with 5 or 20 μmol/L N6L. **A.** PSA and AR expressions were analyzed by Western blot. **B.** Densitometry quantification (ImageJ software) of protein expressions in LNCaP cells treated or not with N6L for 48 hours showed by protein/β-tubulin ratios density. Means ± sd (n = 3), * p < 0.05. **C.** mRNA expression of PSA (KLK3) measured by qRT-PCR in LNCaP cells treated or not with N6L. Means ± sd (n = 3).

Since the modulation of AR activity by NPM1 could be mediated by NPM1 phosphorylation which can be impaired by N6L, we then investigated N6L effect on AR expression and activity as assessed by the AR target gene product KLK3 (PSA, prostate-specific antigen) expression in LNCaP and VCaP cells. AR expression was slightly downregulated after 20 μmol/L N6L treatment during 48 hours but could not achieve significance after densitometry analysis of Western blots (Figure 3A and 3B). By contrast, after 48 hours of treatment, N6L induced a significant decrease in PSA expression in a dose- and time-dependent manner with reduction about 38% at 5 μmol/L and 48% at 20 μmol/L (Figure 3A and 3B). This downregulation of PSA was confirmed at the RNA level by qRT-PCR in LNCaP cells (Figure 3C). Altogether, these results suggest that N6L inhibits NPM1 phosphorylations on Thr 199 and Thr 234/237 and AR activity.

### N6L inhibited prostate tumor growth *in vitro* and *in vivo* and displayed additive effect with castration

**Figure 4:**
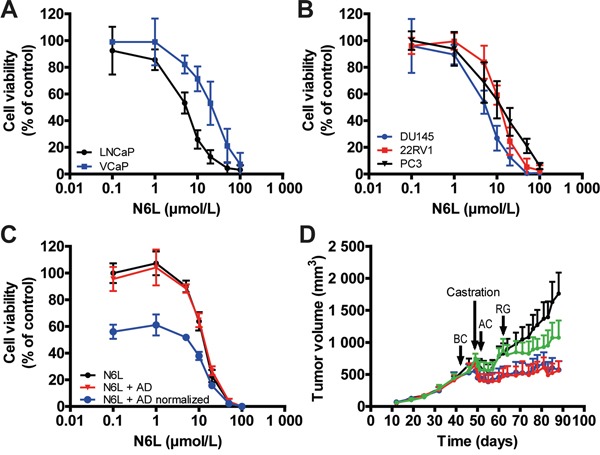
N6L inhibited human prostate tumor cell growth *in vitro* and *in vivo* **A** and **B.** Viability of cells treated for 72 hours with incremental N6L concentrations was evaluated using MTT staining in androgen sensitive (A) or independent (B) cells. **C.** Effect of the combination of N6L and androgen deprived (AD) condition. LNCaP cells were treated for 24 hours with N6L or with PBS and then, incubated in complete medium or in red phenol free RPMI, 10% cs-FBS (AD, mimicking hormonotherapy) for 48 hours. Cell viability was measured using MTT staining. The N6L curve represents the effect of N6L alone, the N6L + AD curve represents the effect of the combination N6L and AD and the N6L + AD normalized curve represents the effect of the combination N6L and AD with a 100% of cell viability corresponding to hormonotherapy treated cells without N6L treatment. **D.** Effect of the N6L/castration combination *in vivo* in VCaP ectopic xenografts in nude mice. Mice bearing tumors of about 600 mm^3^ were castrated. Treatments were performed three times per week by intra-peritoneal injections of PBS (Control group, black curve) or 10 mg/kg N6L one week before castration (BC, blue curve), one day after castration (AC, red curve) or during tumor regrowth (RG, green curve) (n = 8 per group).

The inhibition of NPM1 phosphorylation by N6L leading to a decrease in AR activity highlights its potential for clinical use in PCa. In this context, we evaluated the *in vitro* inhibitory activity of N6L on prostate tumor cell growth using two cell lines considered as androgen-dependent, LNCaP and VCaP, and three as androgen-independent, DU145, 22RV1 and PC3. All cell lines were found to be sensitive to N6L after 72 hours of treatment as assessed using MTT assays (Figure 4A and 4B). The calculated GI_50_ were LNCaP: 5.1 ± 1 μmol/L, VCaP: 22.3 ± 6 μmol/L, DU145: 6.1 ± 1.9 μmol/L, 22RV1: 11.1 ± 4.4 μmol/L and PC3: 10.2 ± 2.9 μmol/L. Since three of the cell lines are androgen-independent, these results suggest that impairment of NPM1 phosphorylation by N6L do not interfere only with AR signaling and demonstrate that N6L use for advanced PCa treatment merits to be considered.

**Figure 5:**
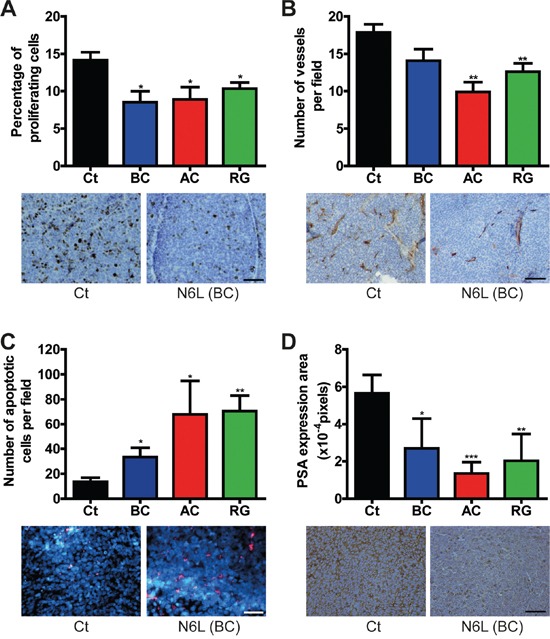
N6L inhibited cell proliferation, angiogenesis and PSA expression and induced apoptosis *in vivo* Tumors from the VCaP ectopic xenografts were removed at the end of the experiment and were analyzed by immunochemistry. Ct represents the control group treated with PBS, BC the group treated with N6L one week before castration, AC the group treated one day after castration and RG the group treated during regrowth. For each analysis, quantification is represented on the top panel. Means ± sd (n = 8), * p < 0.05, ** p < 0.01, *** p < 0.001. Illustrating pictures from the control group and the group treated with N6L one week before castration are represented below. **A.** Effect of N6L on tumor cell proliferation measured using KI-67 immunostaining. Scale bar, 100 μm. **B.** Effect of N6L on tumor angiogenesis evaluated by CD34 staining. Scale bar, 200 μm. **C.** Effect of N6L on tumor cell apoptosis evaluated using fragmentated DNA staining. Scale bar, 100 μm. **D.** Effect of N6L on PSA expression evaluated using anti-PSA antibody. Scale bar, 100 μm.

In clinical practice, advanced PCa are treated with hormonotherapy. In an attempt to mimic and evaluate hormonotherapy in combination with N6L, LNCaP and VCaP cells were first treated for 24 hours with N6L and then cultured in complete medium or in androgen-deprived medium (cs-FBS). As previously described, when LNCaP cells were switched to culture media containing cs-FBS, cell growth was reduced [35], herein about 43% after 48 hours of androgen deprivation (AD). Simultaneous exposure of cells to N6L and AD showed additive effect (Figure 4C). This was highlighted by the “N6L + AD normalized” curve in which the 100% of cell viability corresponds to cells only treated with AD and where the decrease in cell growth was similar to the one without AD. These results were confirmed using the VCaP cell line (Supplementary Figure 3). According to these *in vitro* results, we next analyzed the combinatory effect on tumor growth *in vivo* using VCaP ectopic xenograft model which mimics clinical response to castration [36]. Once the mean tumor volume reached approximately 600 mm^3^, mice were castrated by surgery in order to block androgen synthesis. As already described [36], in response to castration, tumor volume of control mice decreased for 2 weeks after which tumor became castration-resistant and progressed again independently of androgens as observed in the clinical setting (Figure 4D). N6L treatment resulted in a significant stabilization of tumor growth and an additive effect with castration when this molecule was administrated 1 week before or 1 day after castration. In addition, our results also showed the ability of N6L to block over a 28 days period the growth of castration resistant tumor cells during the regrowth phase. Immunohistological analyses of tumor demonstrated a reduction in tumor angiogenesis (CD34), in proliferative activity (KI-67) and an increase in apoptosis (tunnel) in tumors treated with N6L compared with control tumors (Figure 5). Taken together, these results indicate that N6L inhibits the growth of several PCa cell lines, and displays an additive effect with androgen deprivation *in vitro* and *in vivo*.

### NPM1 and its phosphorylated forms are overexpressed in CRPC

**Figure 6:**
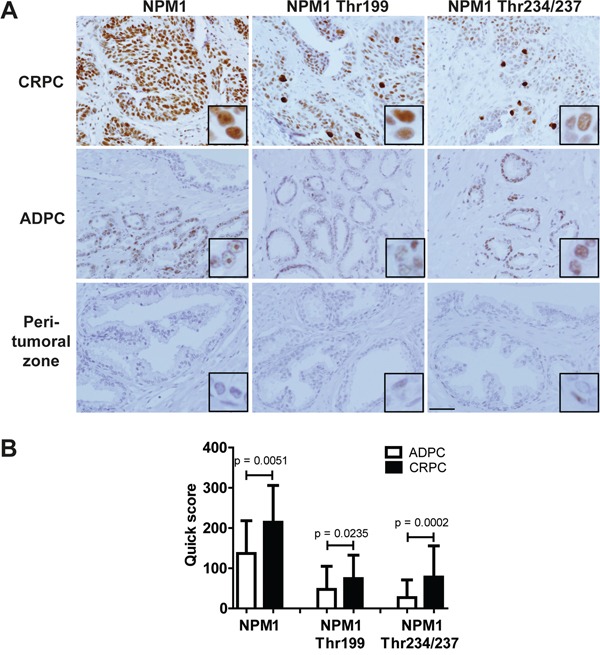
NPM1 and its two Thr199 and Thr234/237 phosphorylated forms were overexpressed in human CRPC 68 human PCa slides were immunostained using NPM1, Thr199 NPM1 and Thr234/237 NPM1 primary antibodies. **A.** Pictures representing the different expressions in castration resistant prostate cancer (CRPC), in androgen-dependent prostate cancer (ADPC) and in normal peri-tumoral zones. Scale bar, 250 μm. **B.** Staining quantifications in CRPC and ADPC using quick scores defined by the intensity and the percentage of stained cells. The p values were obtained using ANOVA unpaired t-test.

Since our results suggest a potent effect of N6L on PCa *in vitro* and *in vivo* and a role of NPM1 phosphorylation in PCa progression, we next assessed the pattern of expression of NPM1 and its phosphorylated forms (Thr199 and Thr234/237) in different stages of human PCa. For this purpose, 20 tissue slides from CRPC and 48 tissue slides from androgen-dependent CaP (ADPC) (11 Gleason 5, 13 Gleason 6, 12 Gleason 7, 6 Gleason 8, 5 Gleason 9 and 1 Gleason 10) were labeled for NPM1, NPM1 Thr199 or NPM1 Thr234/237. NPM1 and its two phosphorylated forms were found at increased levels in tumor cells as compared with that in peri-tumoral normal cells (Figure 6A). In most cases, both NPM1 and its phosphorylated forms were also observed in the normal peri-tumoral zones but at much lower levels, except for NPM1 Thr234/237 where the expression was especially high in the nucleus of basal cells. The three forms were also significantly higher expressed in CRPC tumors than in ADPC (Figure 6A and 6B). In CRPC, NPM1 staining was observed in nucleolus and in nucleoplasm, whereas in ADPC it was only observed in nucleolus. In all cases, NPM1 Thr199 and NPM1 Thr234/237 stainings were observed in nucleoplasm. No significant difference in expression was observed with the Gleason values (≤ 7 compared to > 7), with the total PSA level before surgery (< 10 ng/mL compared to ≥ 10 ng/mL), with the T grade (≤ T2c compared to ≥ T3a) or presence or absence of cancer recurrence. Furthermore, we observed some cells with high expression of both phosphorylated NPM1 forms in all cellular compartments that may correspond to mitotic cells (Figure 6A). These data suggest that NPM1 and its two phosphorylated forms could be important in the progression to CRPC state and represent promising targets for advanced PCa treatment.

### N6L displayed an additive effect with docetaxel chemotherapy *in vitro* and *in vivo*

**Figure 7:**
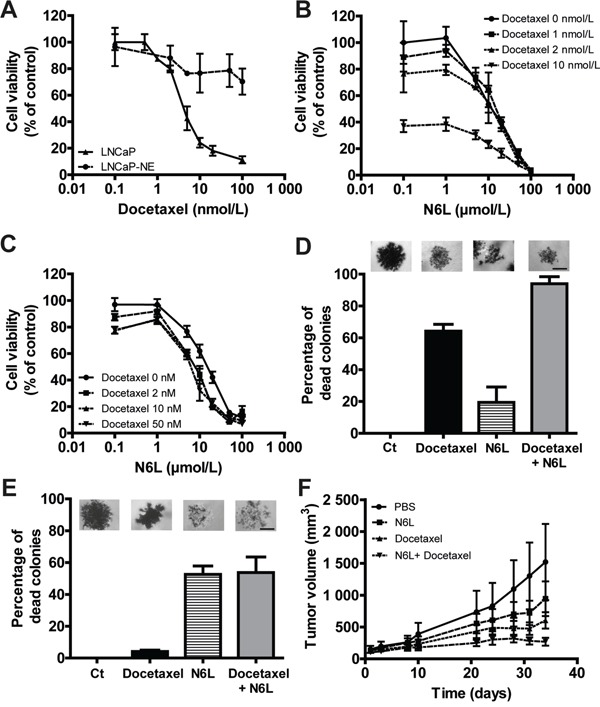
Combination effect of N6L and docetaxel on tumor growth *in vitro* and *in vivo* **A-C.** Cells were treated for 72 hours with N6L and/or docetaxel. Cell viability was then measured using MTT staining. A, Inhibition of LNCaP and LNCaP-NE cell viability with docetaxel treatment. B and C, Effect of the combination N6L/docetaxel on LNCaP (B) and LNCaP-NE (C) cell viability. **D** and **E.** Effect of 50 μmol/L N6L, 100 nmol/L docetaxel and the combination 50 μmol/L N6L/100 nmol/L docetaxel on LNCaP (D) and LNCaP-NE (E) colony viability in soft agar. Cells were treated for 72 hours and colony viability was measured by MTT staining (alive cells appeared in dark/violet). A colony was considered as dead when 50 % of the cells forming the colony were dead (white cells). A picture representing results for each condition is represented above the corresponding graph bar. Scale bar, 100 μm. **F.** Effect of N6L, docetaxel and the combination on tumor growth *in vivo* in DU145 ectopic xenografts in nude mice. Mice bearing tumors of approximatively 100 mm^3^ were treated by intra-peritoneal injections three times per week with PBS (control group) or 10 mg/kg N6L and/or two times per week with 5 mg/kg docetaxel (n = 8 per group).

Castrate resistant PCa patients are currently treated by taxane based chemotherapy (docetaxel). We thus investigated the combined effects of N6L and docetaxel on tumor growth. For this purpose, we evaluated cell viability evaluating by MTT assays (Figure 7A), using the LNCaP cells which are sensitive to docetaxel and LNCaP-NE, which are chemoresistant cells [35]. In accordance with this previous study, LNCaP were sensitive to docetaxel with a GI_50_ of 5 nmol/L whereas LNCaP-NE displayed a resistance to docetaxel with a growth inhibition of 30% at high concentration (100 nmol/L). In LNCaP cultures, the N6L/docetaxel combination resulted in an additive growth-inhibiting effect (Figure 7B). The combination index (CI), obtained using the Chou-Talalay method, was 1.08 ± 0.05 confirming the additive effect. These results were also confirmed on two docetaxel sensitive but androgen-independent cell lines, 22RV1 and DU145 (Supplementary Figure 4) with CI of 0.97 ± 0.28 and 0.92 ± 0.12 respectively. As expected, only the effect of N6L was noted in LNCaP-NE cells (Figure 7C). To confirm these results, the combination was then tested in anchorage-independent conditions. Docetaxel (100 nmol/L) induced death of about 65% in LNCaP derived colonies and 50 μmol/L N6L had only minor effects with 20% dead colonies (Figure 7D). The N6L/docetaxel combination induced 95% of dead colonies, again showing an additive effect of both treatments. With regards to LNCaP-NE, cells were resistant to docetaxel. N6L induced 57 % of colony death and comparable results were obtained for the combination further indicating that only N6L is potent against these cells (Figure 7E). Finally, the combination was tested *in vivo* using the model of DU145 ectopic xenografts in nude mice. Since DU145 cells are androgen–independent and sensitive to docetaxel and N6L, we thought to use this model to evaluate the efficacy of drugs that could be employed to treat patients in the CRPC context. A superior effect was observed when using the combination compared with either drug alone (Figure 7F). All together, these results strongly suggest that a treatment of CRPC with N6L may potentially enhance responses observed with docetaxel chemotherapy alone.

## DISCUSSION

Since current therapies to treat CRPC patients remain insufficient, further research is needed to find new molecular targets and to develop new therapeutic agents.

NPM1 is a multifunctional protein overexpressed in proliferating cells such as tumor cells and has been proposed as a marker of different solid tumors [20–27]. In this study, we found NPM1 to be overexpressed in tumor cells compared to adjacent normal tissue. In addition, we have shown for the first time that two of its phosphorylated forms, Thr199 and Thr234/237, were also overexpressed in tumor cells compared to adjacent normal cells. Previous studies reporting on NPM1 expression in hormone naïve PCa have found either no significant differences [37] or overexpression in tumor cells compared to adjacent normal tissue [19, 32].

Markedly, in this survey, NPM1 and its two phosphorylated forms were found to be significantly upregulated in CRPC compared to ADPC. With regards to prognostic issues, NPM1 has been associated with tumor grading in hepatocellular and endometrial carcinomas but not in oral squamous carcinoma [21, 22, 38]. In this survey, we did not see any association of NPM1 forms with standard clinico-pathological parameters including the pathological grade (as assessed by Gleason score defining prognosis from histopathological values) supporting the idea that NPM1 phosphorylation and overexpression may play a role in castration-resistant phenotype of PCa but not in the progression from low to high grades.

The link between AR signaling and NPM1 in prostate was first demonstrated by an increase in NPM1 expression after androgen stimulation in rat ventral prostatic nuclei [39]. Moreover, a decrease in NPM1 expression and in its phosphorylation was earlier described during apoptosis of epithelial prostate cells in rat after castration [40]. Recent studies have demonstrated that NPM1 could bind to AR and enhanced its transcriptional activity [32]. Since AR is expressed in nucleoplasm and not in nucleolus, which is preferential localization of NPM1, we examined if this interaction could occur in the nucleoplasm where NPM1 is phosphorylated especially at the Thr199 and 234/237 sites. This hypothesis was supported by the fact that the domains involved in the AR/NPM1 interaction are the binding DBD/Hinge of AR with the Hetero D/NBD of NPM1 spanning the amino acids 190 to 292 including the two phosphorylating sites Thr199 and Thr234/237 [32]. We found that phosphorylated NPM1 colocalized with AR in some spots in the nucleoplasm of LNCaP and VCaP cells. Previous studies have reported the localization of similar spots observed for the Thr199 phosphorylated form of NPM1 in mouse skin fibroblast to be nuclear speckles, suggesting pre-mRNA processing activity of NPM1 [33]. These data were confirmed by the colocalization of the two phosphorylated form of NPM1 with the nuclear speckles marker SC35 (Supplementary Figure 1). The role of AR in the nuclear speckles remains unclear but it has previously been shown that AR activity could be modulated in the nuclear speckles after binding with the Ski-interacting protein [41]. The colocalization of two phosphorylated forms of NPM1 with AR suggests their potential role in the regulation of AR activity.

Treatment of LNCaP or VCaP cells with N6L inhibited NPM1 phosphorylation without noticeable change in total NPM1 expression. This inhibition was due to a general decrease in expression as well as a reduced number of strongly positive cells during mitosis. Additional analysis of the LNCaP cell cycle by FACS after BrdU and PI staining showed that N6L blocked LNCaP cells in G2/M phase with a strong decrease in the S phase (Supplementary Figure 5). These two results suggest a blocking of the LNCaP cell cycle in the G2 phase which is in agreement with the role of Thr199 phosphorylated NPM1 for centrosome dissociation during the initiation of cell duplication [14]. Decrease in NPM1 phosphorylation was associated with an inhibition of PSA expression reinforcing the hypothesis that the Thr199 and Thr234/237 phosphorylated form of NPM1 could participate to AR activity. Our results highlight that NPM1 and its phosphorylated forms were overexpressed in CRPC and could participate to AR signaling, supporting the hypothesis that NPM1 may play important roles in resistance to castration and pinpoint the NPM1 as a promising target for CRPC therapy.

Our *in vitro* and *in vivo* experimental studies revealed N6L anti-tumoral activity in numerous androgen-dependent or -independent human PCa cell lines. All these cell lines were sensitive to N6L with no correlation with the androgen-dependent or -independent state of the cells implying that the effect of N6L on human prostatic tumor cells is not solely due to the inhibition of AR activity. NPM1 is a protein involved in many cellular processes including chaperon activity and ribosome biogenesis, but also in apoptosis and cell cycle. It could block the degradation of P53 and ARF acting as an anti-apoptotic molecule [42] [43] or cell cycle where the inhibition of its expression lead to cell cycle arrest in G2/M phase [44]. Recently, it has been shown that NPM1 could favor migration, invasion and colony forming of prostate tumor cells [45]. NPM1 blockade and inhibition of its phosphorylation could disrupt all these processes independently of AR activity. Furthermore, N6L could act through several mechanisms involving nucleolin and sulfated glycosaminoglycans [46].

One notable aspect of our observations is the potential of N6L in improving the therapeutic efficacy of androgen deprivation and docetaxel *in vitro* and *in vivo* showing in both cases additive effects. It is noteworthy to mention that certain PCa cells have the capacity to transdifferentiate to acquire a neuroendocrine (NE) like phenotype which has been proposed as a mechanism for chemo and castration resistances [35] Moreover, there has been increasing concerns that with the introduction of novel potent AR-targeted drugs into the clinic for CRPC, the incidence of Neuroendocrine PC (NEPC) may increase [47]. Here, we tested *in vitro* the efficiency of N6L on neuroendocrine-like tumor cells (LNCaP-NE) and found that LNCaP-NE are remarkably sensitive to N6L. This highlights potential effects and novel therapeutic opportunities to prevent and treat NEPC development in combination with anti-AR approaches. Moreover, it has been shown that NE markers such as β-tubulin III and epithelial-to-mesenchymal transition could be responsible for docetaxel resistance [35] [48]. The effect of 48 hours N6L treatment on LNCaP-NE cells on neuroendocrine β-tubulin III and mesenchymal vimentin markers was evaluated by Western blot. No effects were observed which is consistent with an additive and not synergistic effect of the N6L/docetaxel combination treatment (data not shown).

In summary, the data presented herein bring to light the potential implication of the phosphorylated forms of NPM1 in AR signaling and progression towards CRPC state, although its full biological functions independent of AR remain to be elucidated. Using the NPM1 targeting multivalent pseudopeptide N6L we demonstrated that inhibition of NPM1 phosphorylation led to inhibition of AR activity, although the exact mechanism by which N6L inhibits NPM1 phosphorylation remains to be elucidated. These results also highlight N6L as a promising therapeutic for advanced PCa.

## MATERIALS AND METHODS

### Human prostate tissue samples and immunohistochemical analysis

Formalin-fixed paraffin-embedded (FFPE) prostate tissues have been collected as part of an Institutional Review Board-approved protocol at the Department of Pathology at the Henri Mondor Hospital, Créteil, France. Pathological specimens of hormone-naive PCa (n=48) used in this study were obtained from patients treated for localized disease by radical prostatectomy. Primary CRPC specimens (n=20) were collected at the time of the trans-urethral resection of the prostate in patients who experienced disease recurrence after an initial response to hormone therapy. Recurrence was defined as patients having PSA progression despite a complete androgen blockade therapy and a castrated level of testosterone. FFPE tissues were sectioned at 5 μm thickness and deparaffinized. Antigen were unmasked by heat retrieval with pH 6 citrate buffer for 15 min and endogenous peroxidase activity was inactivated with a 3% hydrogen peroxide solution for 10 minutes. Unspecific stainings were blocked using Superblock solution (TermoFisher Scientific, Illkirch, France) for 1 hour at 37°C. Tissues were then immuno-stained overnight at 4°C with one of the following antibodies: anti-nucleophosmin (ab10530, Abcam, Mulhouse, France, 1:5 000), anti-phospho-NPM1 Thr199 and Thr234/237 (respectively 619201 and 619101, Biolegend/Cliniscience, Nanterre, France, 1:150). Immuno-complexes were revealed using the anti-mouse and anti-rabbit ImPRESS reagent peroxidase kits and the DAB substrate (Vector/Clinisciences). Tissues were then stained with hematoxylin and dehydrated. Slides were mounted using Eukitt medium. Protein expression was scored as null (0), weak (1), moderate (2) and strong (3) and the percentage of tumor cells stained was noted. The multiplication of the score and the percentage of stained tumor cells gave the quick score (between 0 and 300). Evaluation was performed separately by one genitourinary pathologist (YA) and by two non-pathologists (DD and FV).

### Cell culture

All cell lines were purchased from ATCC (American Type Culture Collection/LGC Promochem, Molsheim, France). 22RV1, DU145 and PC3 cells were cultured in RPMI supplemented with 10% of fetal bovine serum (FBS). LNCaP were cultivated in RPMI, 10% FBS, 2 nmol/L dihydrotestosteron (DHT) and VCaP in DMEM, 20% FBS, 2 nmol/L DHT. LNCaP-NE cells were obtained from LNCaP cells cultivated in red phenol free RPMI, 10% cs-FBS (charcoal/dextran-treated serum) for 2 weeks to 1 month. All culture reagents were purchased from Life Technologies (Saint Aubin, France).

### Immunofluorescence

Cells (1.5x10^5^) seeded on cover glass in 24-wells plates were starved for 24 hours, stimulated with 10 nmol/L DHT for 1 hour and treated or not with N6L as mentioned. Cells were fixed with cold methanol for 10 minutes and saturated (3% goat serum) for 1 hour at room temperature. Primary antibodies were incubated overnight at 4°C: anti-AR, sc-7305 and sc-816, Santa-Cruz (Heidelberg, Germany, 1:400) anti-NPM1, ab10530, Abcam (1:500), anti-NPM1 Thr199 and Thr234/237, respectively 619201 and 619101, Biolegend (1:200) and secondary antibodies coupled to Alexa-Fluor 488/555, A11029, A11034, A21424, A21429, Molecular Probes/Clinisciences (1:1000) for 1 hour at room temperature. Nuclei were labeled with 1 μg/mL DAPI. Cover slips were mounted with Mowiol mounting solution. Confocal fluorescence images were acquired using an IX81 inverted Olympus microscope equipped with a DSU spinning disk confocal system (Olympus, Rungis, France), coupled to an OrcaR2 CCD camera (Hamamatsu Corporation, Massy, France) and were processed as previously described [49]. Briefly, cells were analyzed by acquiring axial z stacks of confocal images (8 μm from the base to the top, with steps of 0.5 μm). Residual blurring was removed by spatial deconvolution. The two antibodies anti-phosphorylated NPM1 from Biolegend were characterized for their specificity to target phosphorylated NPM1 (Supplementary Figure 1A and 1B).

### Immuno-precipitation

Nuclear proteins were extracted using the NE-PER Nuclear and Cytoplasmic Extraction Reagent (ThermoFischer Scientific). AR was immuno-precipitated using 200 μg of nuclear proteins and 2 μg of AR antibody (sc-7305, Santa-Cruz) at 4°C overnight. Immuno complexes were recovered using magnetic beads (Bio-ADEMTECH, PESSAC, France).

### Sample preparation, western blot and RT-PCR analyses

Cells (5x10^5^) were seeded in 6-well plates, incubated 24 hours for adhesion and then treated or not with N6L as indicated. For Western blotting, cells were lysed in RIPA buffer and proteins were processed as previously described [31]. Primary antibodies used were: anti-nucleophosmin (1:1 000), anti-NPM1 Thr199 and Thr234/237 (1: 500) and anti-AR (1:2 000) antibodies were the same as for immunofluorescence experiments, anti-PSA (A0562, Dako, Courtaboeuf, France 1:20 000) and anti-β-tubulin (ab6046, Abcam, 1:1 000).

RT-PCR experiments were performed as previously described [35].

### Cell viability assays

Cells (1x10^4^) were seeded in 96-multiwell plates, incubated 24 hours for adhesion and treated as indicated in 5% FBS medium. Cell viability was measured after 72 hours of treatment using the 3-(4,5-dimethylthiazol-2-yl)-diphenyltetrazolium bromide dye (MTT) method (Sigma, Saint Quentin Fallavier, France). GI_50_ values were calculated using the standard curve method. Combination index (CI) were calculated with the Chou-Talalay method using the calcusyn software (Cambridge, United Kingdom). The CI value between 0.95 and 1.05 was interpreted as suggestive of additivism, and >1.05 or <0.95 was suggestive of antagonism or synergism, respectively.

### Colony formation in soft agar

Colony formation was assessed as previously described [50]. For LNCaP-NE colonies, when LNCaP colonies reached 100 μm of diameter, media were changed by cs-FBS media for 10 days and then the colonies were treated.

### Subcutaneous tumor cell xenografts in nude mice

In the N6L/castration combination experiment, VCaP cells (2x10^6^ in PBS, 50 % Matrigel) were injected subcutaneously in the right flank of four-week old male NMRI nude mice. When the tumor reached about 400 mm^3^, mice were separated randomly in several groups. Mice bearing tumors of about 600 mm^3^ were then castrated by surgical testicular removal. Treatments were performed as indicated. In the N6L/docetaxel combination experiment, DU145 cells (5x10^6^) were injected subcutaneously in the right flank of four-week old female NMRI nude mice. When the tumor reached about 100 mm^3^, mice were separated randomly in several groups. Treatments were assessed as indicated. Tumor volume was measured as previously described [31]. All *in vivo* experiments were carried out under the conditions established by the European Community. Tumor analyzes are described in the Supplementary Methods.

### Immunohistochemical analysis on VCaP tumors

Immediately after surgical resection, VCaP tumors were frozen in liquid nitrogen and stored at −80°C or were fixed in formalin, included in paraffin and store at 4°C. For apoptosis analysis, fragmented DNA on frozen slides was stained using the ApopTag Isol Dual Fluorescent kit (Millipore, Saint Quentin en Yvelines, France) as previously described [31]. For angiogenesis, proliferation and PSA expression analyses, tumors were sectioned at 5 μm thickness and deparaffinized. Antigen unmasking was performed by heat retrieval with 10 mmol/L EDTA pH 8 buffer for 20 minutes and endogenous peroxidase activity was inactivated with a 3% hydrogen peroxide solution for 10 minutes. Unspecific stainings were blocked using Power Block Universal reagent (Biogenex Laboratories/Microm Microtech, Francheville, France) for 10 min at 37°C. Tissues were then incubated 2 hours at room temperature with anti-CD34 antibody (rat monoclonal, HM1015, Clinisciences, 1:20), or anti-KI67 antibody (Mouse monoclonal, M7240, Dako, 1:50) or PSA antibody (rabbit polyclonal, A0562, Dako, 1:1,000). Immuno-complexes were revealed using HRP conjugated secondary antibodies and the DAB substrate. Tissues were then stained with hematoxylin and slides were mounted using Mowiol medium. Quantification of peroxidase positive objects (KI67 and CD34) was performed as previously described [49]. PSA images were analyzed as follows: labeling intensity corresponding to cells visually considered as weakly stained was used as threshold level. Objects which staining was higher than this value were then detected and measured.

Numeration of positive apoptotic cells was performed on fluorescent composite images with an original plugin running on ImageJ [50]. Channel corresponding to fragmented DNA labeling was first normalized, and then submitted to a series of filters in order to reduce noise, and to highlight only objects whose size corresponds to cells. This filtering was achieved by a gamma function (1.5) allowing to increase the intensity range of objects which can be detected followed by an unsharp filter (radius 40, weight 0.8) and a fast Fourier transform band pass filter (10-100 pixels). At this step, the transformed image consisting in smoothed objects were then segmented by thresholding using the so called « Triangle » method [51]. Grouped objects were then sub-segmented using the watershed method. To finish, individual objects were submitted to a sorting based on their Signal/Noise ratio. This ratio was calculated for each object as follows: the « Signal » value corresponds to the maximum grey level encountered in the object area projected onto the denoised initial image. « Noise » corresponds to the minimal grey level encountered in the outline of the same projection, added to a correction value equal to 0.5 x SD. Objects were then considered as significantly stained when their Signal/Noise ratio was greater than 1.8. An example of the quantification is shown on the Supplementary figure 6.

### Statistical analysis

Statistical analyses were performed by ANOVA unpaired t test using the GraphPad Prism 4.0 software (San Diego, CA). Values of p < 0.05 were considered significant. Results were expressed as mean ± SD of at least three determinations for each test from three independent experiments.

## SUPPLEMENTARY MATERIALS AND METHODS


