# Supplementary material for: Implication of NPM1 phosphorylation and preclinical evaluation of the nucleoprotein antagonist N6L in prostate cancer
Source: Oncotarget. 2016 Mar 14;7(43):69397–411. doi: 10.18632/oncotarget.8043 (PMC5342486; doi:10.18632/oncotarget.8043)
Supplement: Supplementary file 1 [file oncotarget-07-69397-s001.pdf]

Implication of NPM1 phosphorylation and preclinical evaluation of the nucleoprotein antagonist N6L in prostate cancer

SUPPLEMENTARY MATERIALS AND METHODS

Characterization of the two anti-phosphorylated NPM1 antibodies

Silencing of NPM1 was performed by siRNA experiment as previously described (Destouches et al., 2011).

For alkaline phosphatase treatment, LNCaP cells were starved for 24 hours and then stimulated with 10 nmol/L DHT for 1 hour. Cells were fixed with methanol for 10 minutes at -20°C, air-dried, and re-hydrated in PBS for 10 minutes at room temperature. Cells were incubated in 100 mM glycine, pH 10.4, containing 10 units of alkaline phosphatase type IV (Sigma) for 2 hours at 37°C. The control cells were incubated in the solution without alkaline phosphatase. Cells were washed two times with PBS, immunostained and analyzed by spinning disk fluorescence microscopy.

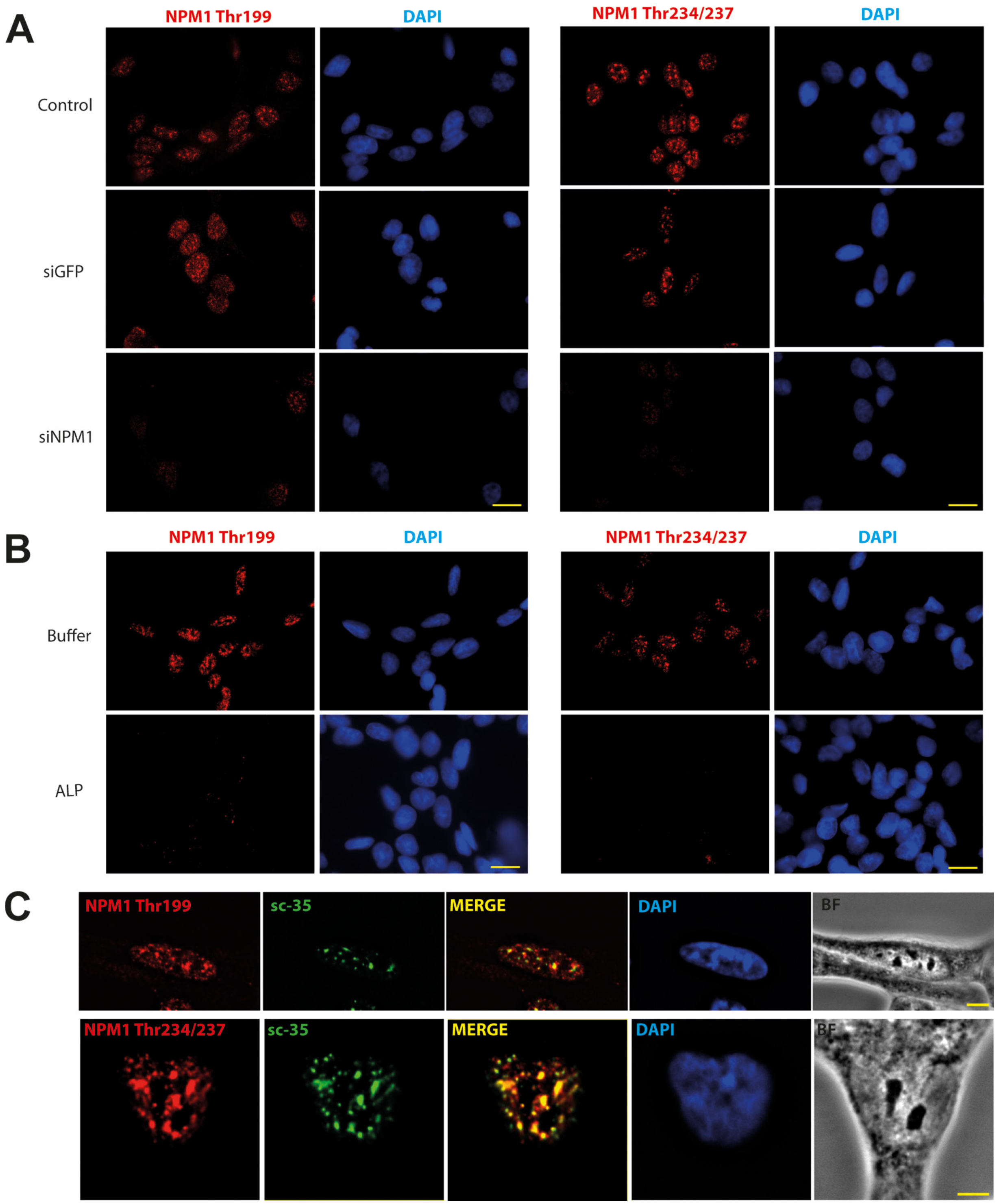

Supplementary Figure S1: Characterization of the two anti-phosphorylated NPM1 antibodies. LNCaP cells were starved for 24 hours and then stimulated with 10 nmol/L DHT for 1 hour. Cells were fixed with methanol, immunostained and analyzed by spinning disk fluorescence microscopy. Nuclei were stained with DAPI. **A.** Before the stimulation with DHT, LNCaP cells were transfected by NPM1 siRNA (siNPM1) or by non targeting siRNA (siGFP) and incubated two days. Control cells were just incubated with the lipofectamin reagent. Scale bars, 20 µm. **B.** Before the immunostaining, cells were treated with alkaline phosphatase in order to dephosphorylate proteins. The control cells (Buffer) were incubated in the solution without alkaline phosphatase. Scale bars, 20 µm. **C.** Cells were immunostained with sc-35 antibody in order to observe the nuclear speckles. Scale bars, 5 µm.

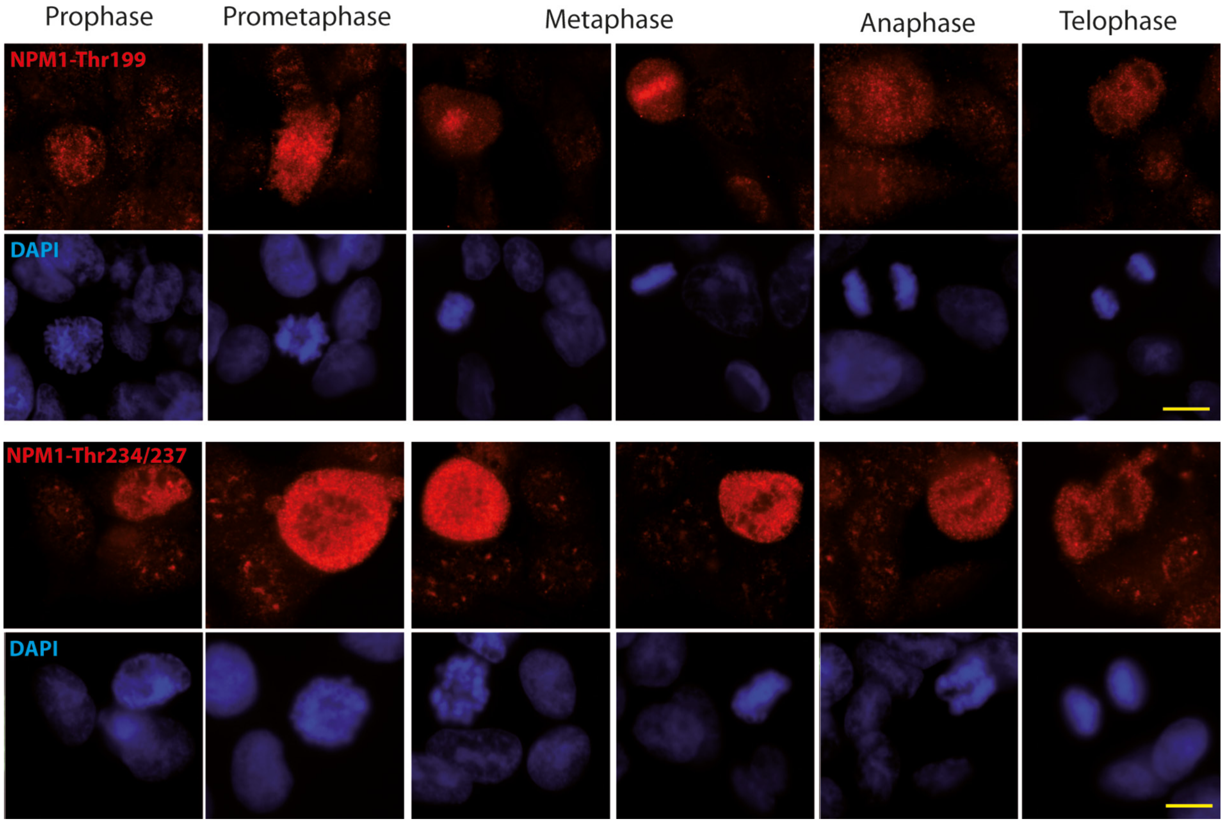

Supplementary Figure S2: Expression of phosphorylated Thr199 and Thr234/237 forms of NPM1 in mitotic LNCaP cells. LNCaP cells were seeded on cover slides in 24 wells-plates for 24 hours in complete medium, starved for 24 hours and were stimulated with 10 nM DHT for 1 hour. Cells were fixed with methanol, immunostained and analyzed by spinning disk fluorescence microscopy. Nuclei were stained with DAPI. Scale bars, 2.5 μm.

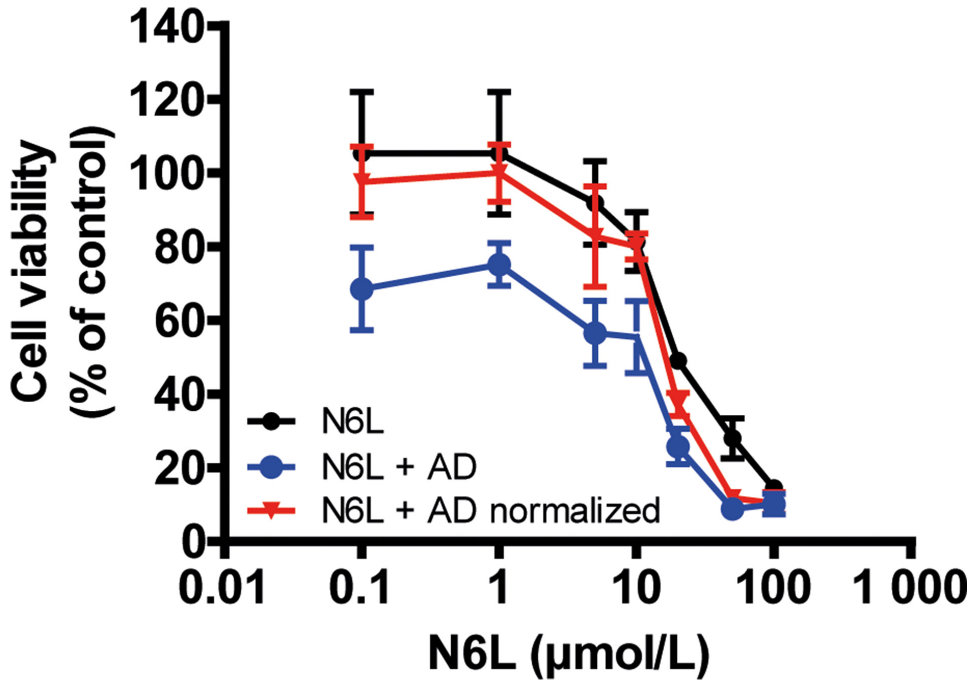

Supplementary Figure S3: Effect of the combination of N6L and androgen deprived (AD) condition on VCaP cells growth. VCaP cells were seeded in 96 wells-plates and treated for 24 hours with N6L at concentration from 0.1 to 100 µmol/mL. To mimic clinical situation in which patients receive hormonotherapy, media were then replaced by red phenol free RPMI, 5 % cs-FBS. Cells were incubated for 48 hours and cell viability was measured using MTT staining. The N6L curve represents the effect of N6L alone, the N6L + AD curve represents the effect of the combination N6L and hormonotherapy and the N6L + AD normalized curve represents the effect of the combination N6L and hormonotherapy with a 100 % of cell viability corresponding to hormonotherapy treated cells without N6L treatment.

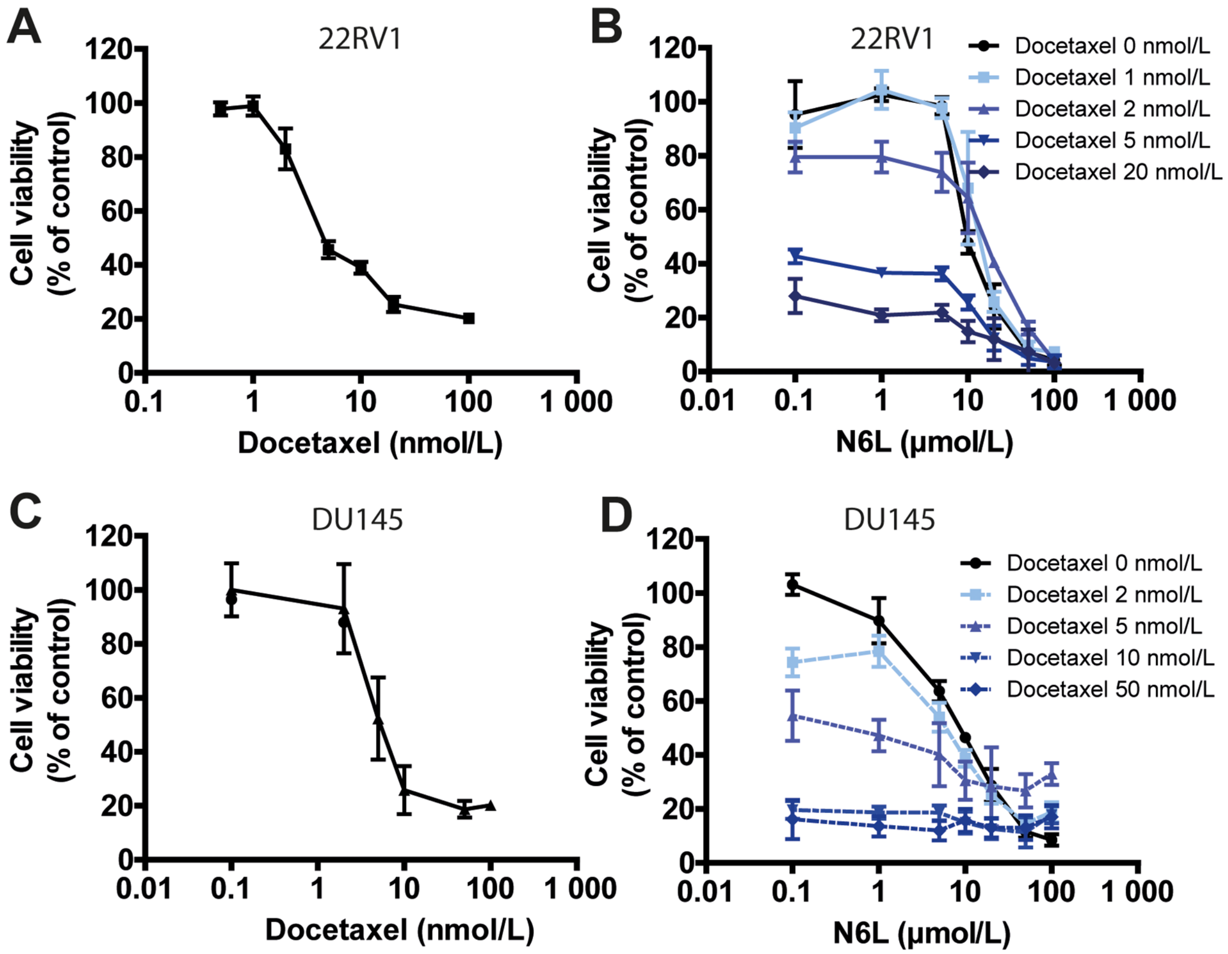

Supplementary Figure S4: Combination effect of N6L and docetaxel on tumor growth *in vitro*. Cells were seeded in 96 wells-plates and were treated for 72 hours with N6L and/or docetaxel. Cell viability was then measured using MTT staining. **A** and **C.** Inhibition of 22RV1 (A) and DU145 (C) cell viability with docetaxel treatment. **B** and **D.** Effect of the combination N6L/docetaxel on 22RV1 (B) and DU145 cell viability (D).

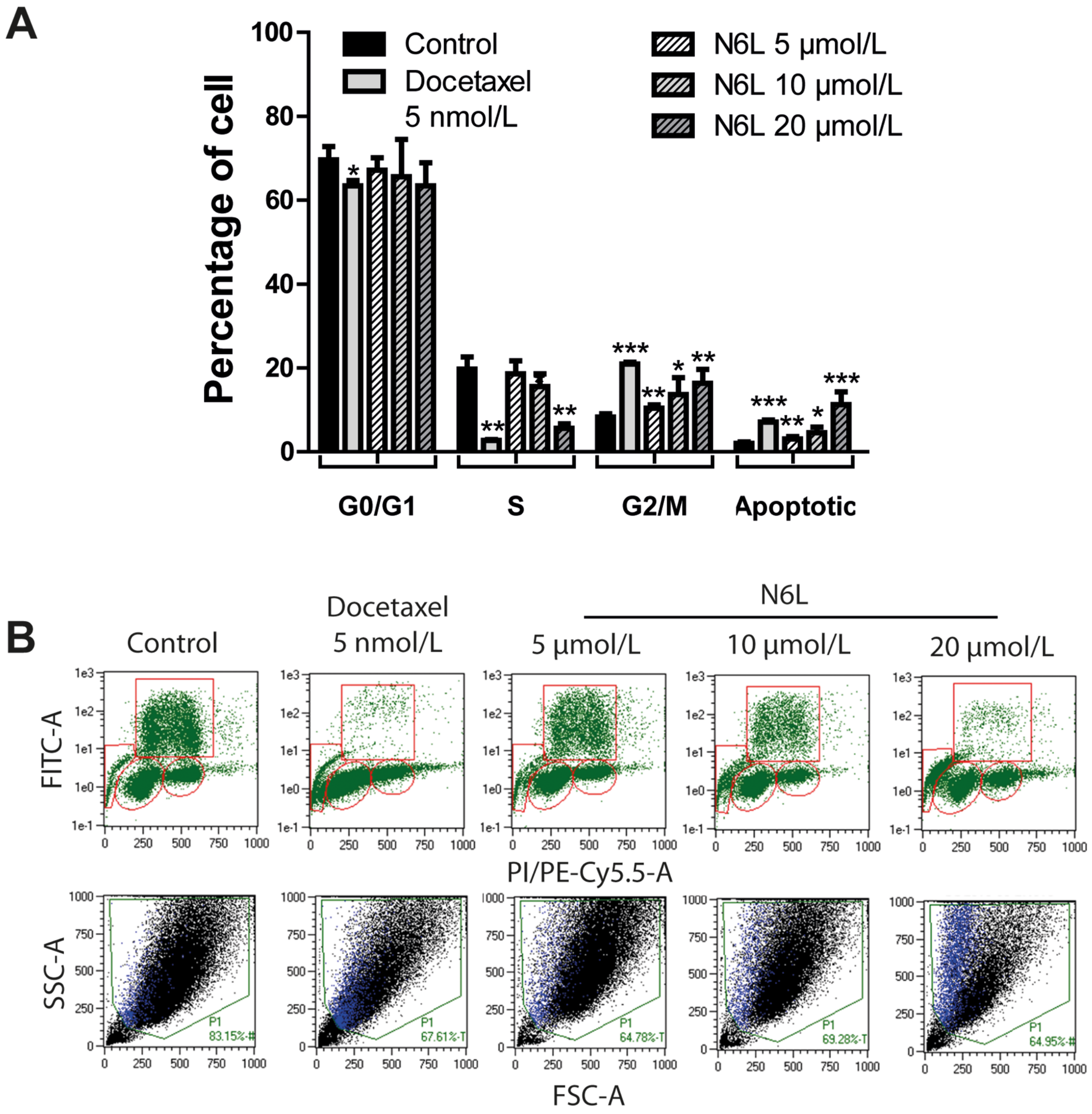

Supplementary Figure S5: Effect of N6L on cell cycle of LNCaP cells. LNCaP cells (5x10^{5}) were seeded in 6 wells-plates and incubated for 24 hours for adhesion. Cells were treated with N6L or docetaxel for 48 hours. Cells cycle was then evaluated using BrdU-PI staining (FITC BrdU Flow Kit, BD Pharmingen) and analyzed by flow cytometry. **A.** Percentage of cells in the different phases of cell cycle. **B.** Representative plots obtained in each condition. The blue points correspond to cells in the apoptotic gate.

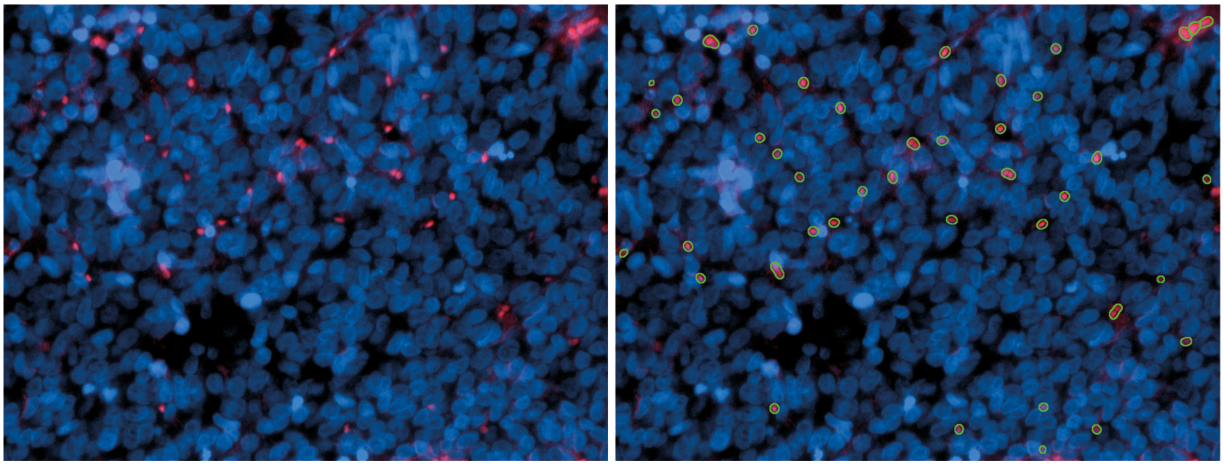

Supplementary Figure S6: Method for fragmentated DNA quantification from immunocyto-fluorescence pictures. Picture showing the original image of fragmentated DNA labeling (left) and after positive cells detection (right). In each picture nuclei are stained with DAPI.
